# Practical Notes and Formulæ

**Published:** 1882-07-20

**Authors:** 


### PRACTICAL NOTES AND FORMULÆ.

**A Common Error that should be Corrected.**—G. G. Roy, M. D., Professor of Materia Medica in Southern Medical College, Atlanta, Ga., sends us the following practical hint, which it is well to observe:

We frequently see in print formulæ thus expressed: (Copied from the Medical and Surgical Reporter, Philadelphia.)

℞ Creasoti, . . . . . . . . . . . . . . . . . . . . . . . . . *m* xx ad f. ʒss,

Zinci oxidi, . . . . . . . . . . . . . . . . . . . . . . . ʒiss,

Ungt. simp. benzoat, . . . . . . . . . . . . . . . ℥i. M.

Now, the books teach, and we teach in our classes, that a *minim* (m) is equivalent to *two drops;* and young graduates (and many of the older) who have been so instructed, might very naturally make a serious, if not a fatal mistake in prescribing poisonous liquid medicines, if the druggist gave the value of the *minim* as equivalent to *two drops*, when it was intended to represent *one drop;* or the patient might suffer, and the physician be disappointed in the effect or want of effect of the medicine, if he intended the *minim* as 2 drops and the druggist used one.

It is true that in the above formula the "ad. f. ʒss" shows that the minim is intended to represent *a drop*—since, if representing *two, forty drops* would be used, which is more than half a fluid drachm—but it is not always thus written. The *minim* is rarely used with us, and the mistake not likely to occur with our druggists; but in using a prescription, which has proven valuable in the hands of other physicians, where the *minim* is directed instead of "*gtt*"—the physician borrowing it would not know which was intended (unless the formula is similar to the one quoted above) and of course could not enlighten the druggist.

The *minim* should be used only to represent *two drops*, (the books teach this) or it would be best to write "*gtt*" altogether, as all know that this symbol means *a drop.*

Uniformity in a matter apparently so simple as this, is nevertheless very desirable, and may sometimes prevent serious, if not fatal consequences to the patient, and relieve the physician and druggist, to say the least, of much severe criticism and even criminality.

**Treatment of Uterine Fibroids.**—Fibroids of the uterus may often be successfully treated by the use of suppositories of ergotin made according to the following formula:

Ergotin . . . . . . . . . . . . . . . . . . . . . . . . . . . gr. 1-12; 0.006 Gm.;
Cacao butter . . . . . . . . . . . . . . . . . . . . . . gr. xxiij; 1.50 Gm.;
Vaselin . . . . . . . . . . . . . . . . . . . . . . . . . . . q. s.

For one suppository.

These suppositories are of equal use in cases of menorrhagia, metrorrhagia, and chronic metritis.—*Le Prog. Med.; Lon. Prac.*

**Chronic Ulcer.**—Dr. Erichsen, in Detroit Clinic, reports a case of chronic ulcer cured as follows—

John G., aged 58. This man had a small ulcer on his right leg. No specific history. The cause could not be ascertained. His general health was good. I prescribed—

℞ Iodoformi, .......... ½ drachm,
Balsam Peru, .......... q. s,
Cerati simp .......... 1 ounce.
M. Ft. unguentum.

Sig. To be applied to ulcer night and morning.

Improvement took place rapidly, and in five weeks the man was relieved of his trouble.

**Comedone Wash**—

Cologne .......... 100 parts,
Benzoic acid .......... 5 parts,
Balsam Peru .......... 1 part,
Mix and add :
Alcohol .......... 300 parts,
Conc. acet. acid .......... 500 parts.

ANOTHER ONE.

Ac. oxalic .......... 5 parts,
Borax .......... 10 parts,
Rosewater .......... 135 parts,
Glycerine .......... 50 parts.

These might replace the secret or patented Comedone washes. —*New Idea.*

**New Treatment for Vaginitis.**—M. Terrillon proposes a method of treatment which consists essentially in the introduction into the vagina of the following ointment—

℞ Ac. tannic .......... 50 grams,
Amyli .......... 150 grams,
Ung. petrolei .......... 150 grams.

M. This ointment is placed in a sort of speculum, so arranged that the ointment can be forced out as the instrument is withdrawn from the vagina. If the vulvar opening is large a small tampon of cotton may be introduced. Generally from fifteen to twenty grams of the unguent is sufficient at one application, and it need not be repeated for seven or eight days.—*Med. and Surg. Rep.*

**Mercurial Salivation.**—For the prevention of it, Professor Panas prescribes the following—

Powdered cinchona .......... 3 parts,
Powdered rhatany .......... 1 part,
Powdered chlorate of potash .......... 1 part.

Rub the gums ten or twelve times a day.—*Ibid.*

**Camphorated Dover Powders.**—Dr. A. H. Chenoweth, of Olney, Missouri, says: I have been using for three or four years an anodyne and diaphoretic powder of my own invention, made after the following:

| | | |
|---|---|---|
| R | Powdered opium | ½ scruple, |
| | Powdered ipecac | 1 scruple, |
| | Powdered camphor | 2 scruples, |
| | Powdered potassa bromidi | 8 scruples. |

Throw together in a mortar, and rub into a fine powder. Dose, the same as the old powder.—*Brief.*

**Treatment of Amenorrhœa.**—A large number of remedies have been credited with emmenagogue properties, many of them being inert, and some of them simply irritant poisons whose employment has frequently resulted fatally, especially when used with criminal intent, as abortifacients. Strychnia affords excellent results in many instances. A favorite with me is the following—

| | | |
|---|---|---|
| R | Strychniæ sulph | gr. j, |
| | Quiniæ | ʒ j, |
| | Ferrum per hydrogen | aa ʒ ij, |
| | Asafetidæ pulv | (aa ʒ ij) |
| | Ext. qussiæ | q. s. |

In pil. No. 60 div. Sig. One four times daily.

I usually add at bed-time ten drops of Squibb's fluid ext. ergot in water; and a forcible jet of cold water along the spine every morning on rising for a few minutes, with brisk friction of the abdomen, succeeds admirably in many cases. Exercise in the open air, equestrianism particularly, with attention to a normal action of the skin, kidneys, and bowels is essential.—*Med. Summary.*

**Ring Worm.**—

| | | |
|---|---|---|
| R | Adipis | 6 drachms, |
| | Glycerinæ | 2 drachms, |
| | Sodii carbonat | 1 drachm, |
| | Calcis pulv | ½ drachm, |
| | Carbonis (liq.) pulv | 1½ ounces. |

M. Sig. Before applying this salve remove scabs by using starch poultices. Treatment should be kept up two or three months in cases of tinea tonsurans.—*N. O. Med. Jour.*

**Chordee.**—

| | | |
|---|---|---|
| R | Chloral hydrat | ½ drachm, |
| | Camphoræ, | 12 grains, |
| | Morph. acetatis | 2 grains, |
| | Ol. theobroma | q.s. |

M. Ft. suppos. no. 6 (15 grains each).

Sig. One every hour in rectum until relieved.—*Med. and Surg. Reporter.*

